# Associations of Mortality with Long-Term Exposures to Fine and Ultrafine Particles, Species and Sources: Results from the California Teachers Study Cohort

**DOI:** 10.1289/ehp.1408565

**Published:** 2015-01-23

**Authors:** Bart Ostro, Jianlin Hu, Debbie Goldberg, Peggy Reynolds, Andrew Hertz, Leslie Bernstein, Michael J. Kleeman

**Affiliations:** 1 Air Pollution Epidemiology Section, California Office of Environmental Health Hazard Assessment, Oakland, California, USA; 2 Department of Civil and Environmental Engineering, University of California, Davis, Davis, California, USA; 3 Cancer Prevention Institute of California, Berkeley, Berkeley, California, USA; 4 City of Hope National Medical Center, Duarte, California, USA

## Abstract

**Background:**

Although several cohort studies report associations between chronic exposure to fine particles (PM_2.5_) and mortality, few have studied the effects of chronic exposure to ultrafine (UF) particles. In addition, few studies have estimated the effects of the constituents of either PM_2.5_ or UF particles.

**Methods:**

We used a statewide cohort of > 100,000 women from the California Teachers Study who were followed from 2001 through 2007. Exposure data at the residential level were provided by a chemical transport model that computed pollutant concentrations from > 900 sources in California. Besides particle mass, monthly concentrations of 11 species and 8 sources or primary particles were generated at 4-km grids. We used a Cox proportional hazards model to estimate the association between the pollutants and all-cause, cardiovascular, ischemic heart disease (IHD), and respiratory mortality.

**Results:**

We observed statistically significant (*p* < 0.05) associations of IHD with PM_2.5_ mass, nitrate, elemental carbon (EC), copper (Cu), and secondary organics and the sources gas- and diesel-fueled vehicles, meat cooking, and high-sulfur fuel combustion. The hazard ratio estimate of 1.19 (95% CI: 1.08, 1.31) for IHD in association with a 10-μg/m^3^ increase in PM_2.5_ is consistent with findings from the American Cancer Society cohort. We also observed significant positive associations between IHD and several UF components including EC, Cu, metals, and mobile sources.

**Conclusions:**

Using an emissions-based model with a 4-km spatial scale, we observed significant positive associations between IHD mortality and both fine and ultrafine particle species and sources. Our results suggest that the exposure model effectively measured local exposures and facilitated the examination of the relative toxicity of particle species.

**Citation:**

Ostro B, Hu J, Goldberg D, Reynolds P, Hertz A, Bernstein L, Kleeman MJ. 2015. Associations of mortality with long-term exposures to fine and ultrafine particles, species and sources: results from the California Teachers Study cohort. Environ Health Perspect 123:549–556; http://dx.doi.org/10.1289/ehp.1408565

## Introduction

Several cohort studies have reported associations of long-term exposure to fine particles (PM_2.5_; particulate matter ≤ 2.5 μm in diameter) with cardiovascular mortality (Hoek et al. 2013; Laden et al. 2006; Lipsett et al. 2011; Pope et al. 2002). Because PM_2.5_ is a heterogeneous mix of particle sizes and chemistry and is generated from multiple sources, the specific constituents and sources of concern have not been fully elucidated. Until recently, among the constituents of PM_2.5_, long-term exposures (i.e., ≥ 1 year) for cohort studies have been generated only for sulfates and black carbon (Dockery et al. 1993; Pope et al. 1995; Smith et al. 2009). In addition, because of the difficulty in measuring exposure, there has been little focus to date on the health effects of long-term exposure to ultrafine (UF) particles (particles ≤ 0.1 μm in diameter).

Epidemiologic analysis of the effects of particulate matter constituents is hindered by their spatial heterogeneity and the reliance on a few fixed-site monitors to represent exposures in large metropolitan areas. For example, for PM_10_ (particles ≤ 10 μm in diameter), many metropolitan areas have only a small proportion of their total population within 15 km of a monitor, such as New York, New York (3.5%), Detroit, Michigan (23%), Boston, Massachusetts (39%), Seattle, Washington (31%), and Philadelphia, Pennsylvania (35%) [U.S. Environmental Protection Agency (EPA) 2009]. The proportion for those > 65 years of age, a well-documented susceptible subgroup, who are within 15 km are only slightly higher: New York (4%), Detroit (27%), Boston (41%), Seattle (32%), and Philadelphia (38%). Although coverage for PM_2.5_ is much higher given its spatial homogeneity, its constituents are known to be spatially variable and often very localized (Kim et al. 2005). The exposure misclassification will be even greater for measurement of mass and constituents of UF particles given their spatial heterogeneity (Sakurai et al. 2003; Sioutas et al. 2005). Some cohort studies have made use of land use regression (LUR) models to estimate PM_2.5_ or nitrogen dioxide (Beelen et al. 2014) at finer spatial scales, but LUR models for particle sources or species are not widely available.

In a previous study, we examined the relation between mortality and long-term exposure to constituents of PM_2.5_ using data from the California Teachers Study (CTS) cohort (Ostro et al. 2010). Started in 1995, the CTS is a prospective study of > 130,000 current and former female teachers and administrators identified through the State Teachers Retirement System. Because of limited data on particle species, this earlier report relied on PM_2.5_ data collected and further analyzed by the U.S. EPA at eight fixed-site monitors as part of the Speciation Trends Network (U.S. EPA 2008). The 24-hr averaged measurements were usually obtained on an every third- or sixth-day basis. To minimize exposure misclassification, catchment buffer areas of 8 and 30 km were drawn around each monitor. The 30-km buffer is likely too large to capture exposure contrasts of many of the species, while the 8-km buffer significantly reduced the sample size, resulting in more unstable estimates and reduced statistical power. Although these buffers were an improvement over studies using a single or multiple monitors to represent exposure over large metropolitan areas, they may not sufficiently measure concentrations of many of the PM constituents, such as elemental carbon (EC) and transition metals that are known to exhibit high spatial variance. Specifically, Hu et al. (2014a, 2014b) reported significant bias for several species of fine and UF particles when comparing the central-site monitor readings applied to the entire metropolitan area population versus our estimated population-weighted concentrations. The latter are derived as the product (both at the 4-km grid scale) of the population and our model-based estimates of the pollutants.

Model estimates are highly correlated (*r* > 0.8) with observations at the monitoring locations. But for the seven major California Metropolitan Statistical Areas that had available data, the estimated population-weighted concentrations for EC were generally lower than central-site monitor predictions, with a maximum bias of –50% in Los Angeles, California, and an average bias of –33%. Although measurement and model predictions were in good agreement at the monitor locations, the bias was introduced by spatial variability around the monitor.

For the present study, we combined data from the CTS with newly developed exposure data generated from the UCD/CIT (University of California Davis/California Institute of Technology) Source Oriented Chemical Transport model. The UCD/CIT chemical transport model uses calculated meteorological fields and emissions estimates for different sources to predict airborne particulate matter concentrations. Particulate matter emissions are assigned a size and composition distribution based on measurements in source-testing experiments. The source-identity of all particulate matter emissions is retained through the simulated atmosphere. In the present study, ground-level mass concentrations for 50 PM constituents were estimated over the major population regions in California at a 4-km resolution for the period of 2000–2007. For many species of fine and UF particles, model predictions are highly correlated with measured values, particularly for longer averaging times (> 2 weeks). For example, correlations were > 0.8 for comparisons between annual modeled and measured concentrations of 10 different PM_2.5_ components for five of the seven metropolitan regions with available monitoring data (Hu et al. 2014b).

Below, we report our findings of an analysis of the associations of long-term exposure to 19 constituents and sources of both PM_2.5_ and UF particles on mortality from all natural causes, cardiovascular disease, ischemic heart disease (IHD), and pulmonary disease.

## Methods

*Data*. The CTS is a prospective study of 133,479 current and former female teachers and administrators who completed baseline questionnaires mailed to them in 1995 to investigate the incidence of breast cancer in public school teachers and administrators, as described in detail by Bernstein et al. (2002). Subsequent questionnaires were mailed to CTS participants in 1997 and 2000. The design and ongoing follow-up of the CTS cohort is a multi-institutional collaboration involving researchers with diverse and complementary areas of expertise. Record linkage is conducted annually to mortality files administered by the California Department of Public Health. In addition, residential addresses of each CTS participant were updated annually for the mailing of newsletters. The mean age of CTS participants at enrollment was 54 years, with 90% between 30 and 80 years. The cohort is multi-ethnic but primarily non-Hispanic white (86.7%) and born in the United States (93.6%). For this study, we used cohort follow-up data from January 2001 through July 2007. Women < 30 years of age at the start of the study were excluded to focus on mid-life and older women. Use of data on human subjects in the main CTS cohort study was reviewed and initially approved by the California Committee for the Protection of Human Subjects, Health and Human Services Agency, and by the institutional review boards (IRB) for each participating institution in June 1995 and annually thereafter. Informed consent was obtained upon entry into the cohort. Analysis for this manuscript was approved in August 2013 by the IRB of the Cancer Prevention Institute of California, the center of one of the principal investigators.

*Health outcomes*. In this analysis, we focused on associations between long-term exposures and mortality. Deaths were assigned codes based on the *International Classification of Diseases, 10th Revision* (ICD-10) for the following outcomes: all-cause deaths excluding those with an external cause (A00–R99), cardiovascular deaths (I00–I99), IHD deaths (I20–I25), and pulmonary deaths (C34, J00–J98). Person-days at risk were calculated as the number of days starting from 1 January 2001 until the earliest of three dates: *a*) the date of death; *b*) a move out of California for at least 4 months; or *c*) 31 July 2007, the end of follow-up for this analysis. If a woman moved out of state for < 4 months, exposures during that time were not included in the calculations of the long-term average. Women who died from a cause other than the outcome of interest during the follow-up period were censored at the time of their deaths.

*Air pollution exposure estimates*. The UCD/CIT model was used to estimate ground-level concentrations of 50 PM constituents over the major population regions in California using a 4-km grid resolution for the period from 2000 through 2007 (Hu et al. 2014a, 2014b). A sensitivity analysis conducted at 250-m resolution over Oakland, California (Joe et al. 2014), indicated that 4-km resolution captures 55–70% of concentration variability within the urban area.

Using the extensive emissions inventory in California, the model calculations track the mass and number concentrations of the PM constituents in particle diameters ranging from 0.01 to 10 μm through calculations that describe emissions, transport, diffusion, deposition, coagulation, gas- and particle-phase chemistry, and gas-to-particle conversion (Hu et al. 2014b). The model solves the coupled set of differential equations that describe how atmospheric processes change pollutant concentrations in regularly spaced atmospheric grid cells. Thus, the predicted exposure concentrations primarily reflect the balance between emissions inventories of fresh particles and meteorological fields that drive dispersion and chemical reaction.

Model predictions were saved at hourly time resolution and averaged to longer times as needed. Predicted concentrations were evaluated against ambient measurements at all available locations and times. PM_2.5_ mass predictions had a mean fractional bias within ± 0.3 (meeting accepted performance criteria) at 52 sites of the total 66 sites across California after correcting for bias in the dust emissions because many studies have shown that dust emissions in the current emission inventory are overestimated (Hu et al. 2015). Good correlations between predictions and measurements (*r* > 0.8) were demonstrated for many of the PM_2.5_ and UF species at most of the monitoring stations, particularly for the monthly, seasonal, and annual averages. For example, monthly PM_2.5_ nitrates were correlated with measurements with *r* = 0.76 (15 sites), monthly PM_2.5_ EC was correlated with measurements with *r* = 0.94 (8 sites), and monthly PM_2.5_ concentrations of potassium, chromium, zinc, iron, titanium, arsenic, calcium, manganese, and strontium were correlated with measurements with *r* ≥ 0.8 (5 sites of a total of 8). For EC in the UF range, the correlation was above *r* = 0.9 for 117 available measurements made at 13 locations during nine intensive field campaigns that each lasted several weeks (Hu et al. 2014b). The quality of the model predictions summarized above reflects the accuracy of the emissions inventories that have been refined over three decades in California, the development of reactive chemical transport models that include important aerosol transformation mechanisms, and the development of prognostic meteorological models that allow for long simulations of historical meteorology.

**Figure 1:**
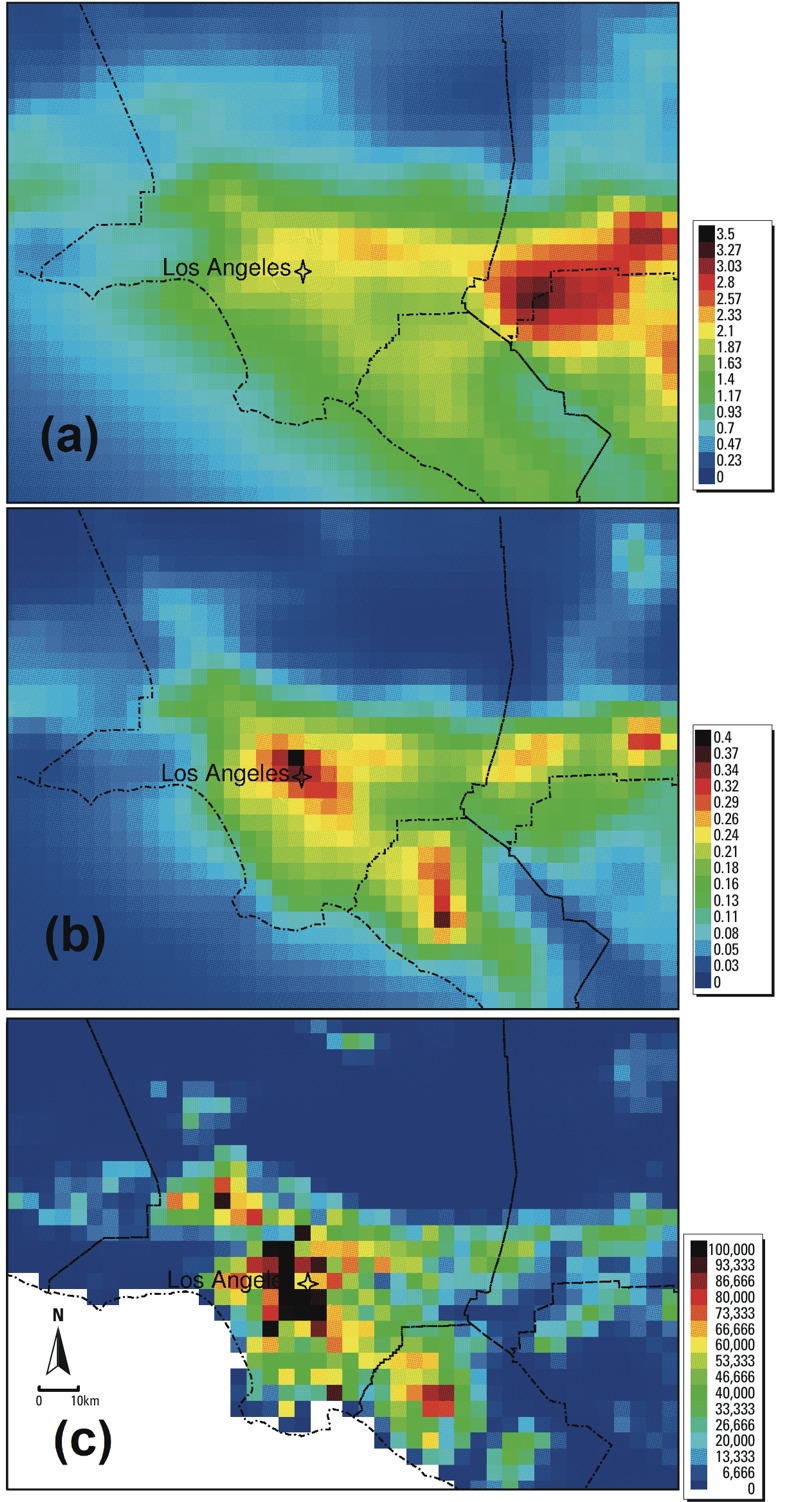
Modeled concentrations (μg/m^3^) of PM_2.5_ nitrate (*A*), ultrafine anthropogenic secondary organic aerosols (*B*), and population in the Los Angeles Basin (*C*) using 4‑km grids (the star in the figures indicates the site of the U.S. EPA monitor).

Coarse particle predictions (2.5 μm < D*p* < 10 μm) have undergone only preliminary comparisons to measurements and were not used for exposure estimates in the present study. Likewise, UF number concentrations were not used because our modeling did not include nucleation, the process by which particles are formed directly from gas molecules, which would greatly impact this parameter. UF mass concentrations are highly correlated with particle surface area (Kuwayama et al. 2013) and serve as a good metric for the potential exposure to UF particles. The measured correlation between UF mass and particle surface was 0.97 in Sacramento, a typical city in central California. For many of the fine and UF species, Hu et al. (2014a) observed strong spatial variability within metropolitan areas (Figure 1).

On the basis of previous studies (Ostro et al. 2007, 2010; Peng et al. 2009; Zanobetti et al. 2009), we chose to examine a subset of the available constituents. Additionally, some constituents were eliminated given their high intercorrelation or low concentrations.

Thus, for each particle size, we analyzed the following constituents: copper (Cu), iron (Fe), manganese (Mn), nitrate, EC, organic carbon (OC), “other” species (i.e., mineral dusts and constituents not measured), “other” metals (those besides Cu, Fe, and Mn that were explicitly resolved), and secondary organic aerosol (SOA). SOA formation was simulated with the mechanism in the U.S. EPA’s Community Multi-scale Air Quality (CMAQ) model version 4.7 (Carlton et al. 2010). SOAs were divided into anthropogenic (SOA_ant: derived from long-chain alkanes, xylenes, toluenes, and benzene and their oligomers) and biogenic (SOA_bio: derived from isoprenes, monoterpenes, and sesiquiterpenes and their oligomers). Nitrate was not estimated for the UF size fraction. Once the constituent concentrations were obtained, Positive Matrix Factorization was used to develop source profiles. Estimates were provided for sources of primary aerosols including on-road gasoline, off-road gasoline, on-road diesel, off-road diesel, wood smoke, meat cooking, high-sulfur fuel combustion (including distillate oil, marine vessel fuel, aircraft jet fuel, liquid and solid waste fuel), and “other anthropogenic.”

Ultimately, the exposure metrics were combined with the updated addresses. Monthly individual exposure estimates were developed through spatial linkage of the geocoded residential addresses. All residences within a given grid in a given month were assigned the modeled pollutant value for that grid for that period. The average long-term pollution exposure for a participant was obtained by calculating the mean of her monthly averages. At the time of each death, the long-term average for each individual remaining in the cohort was recalculated, allowing comparison between the decedent’s long-term average exposure and those of the members remaining in the risk set.

*Covariates*. The individual-level covariates included as explanatory variables in the regression models were based on previous results from air pollution studies for this cohort (Lipsett et al. 2011). Specifically, the covariates included twenty individual-level covariates (a total of 58 terms): age (divided into 2-year categories between 30 and 79 years of age, 3-year categories between 80 and 88 years, and one category for women ≥ 89 years); race [non-Hispanic white, other (African American, Hispanic, Asian, Pacific Islander, and Native American), or unknown]; marital status (married/living with partner, not married, and unknown); smoking status (never, former, and current smokers) and pack-years of smoking (continuous variable for former and current smokers); secondhand smoke exposure (none, household exposure, unknown); body mass index (BMI) (16–19, 20–24, 25–29, 30–39, 40–55 kg/m^2^); lifetime physical activity (tertiles, unknown); alcohol consumption [beer (no/yes/unknown), wine (no/yes/unknown), liquor (no/yes/unknown)]; average daily dietary intake of fat (tertiles, unknown), fiber (tertiles, unknown), and calories (tertiles, unknown); menopausal status and hormone replacement therapy use combined (premenopausal, peri/postmenopausal and no HT use, peri/postmenopausal and past HT use, peri/postmenopausal and current use of estrogen, peri/postmenopausal and current use of estrogen plus progestin, and unknown menopausal status or HT use); family history of myocardial infarction (yes/no) or stroke (yes/no); and use of blood pressure medication (low, medium, high, unknown) or aspirin (low, medium, high, unknown). Data on all individual-level variables except marital status (which was assessed in the 2000 questionnaire) were obtained from the baseline questionnaire.

*Statistical methods*. We fitted Cox proportional hazards models to estimate hazard ratios (HRs) and 95% confidence intervals (CIs) for associations between each pollutant and the outcomes of interest. We examined each pollutant with a separate regression model adjusted for the covariates described above. The Cox model was stratified by age and race/ethnicity. To ensure that we would be examining associations with chronic rather than acute exposures, study exposures began in January 2000, the cohort follow-up began in January 2001, and both continued until July 2007. Two additional sensitivity analyses were conducted. First, we reran the models after including six census-derived contextual (neighborhood) variables including income (median household income), income inequality (percent below poverty level), education (percent with college degree), population size, racial composition (percent white, percent black, percent Hispanic), and unemployment (percent unemployed). These variables were derived from the 2000 census at the block-group level based on the subject’s residence at the time of the baseline questionnaire. These variables represent social, economic, and environmental settings at a group level that may be associated with disease outcomes at the individual level. As such, they may provide additional control for residual confounding. The second sensitivity analysis involved two-pollutant models for a selected set of constituents for the outcome IHD mortality. Specifically, we took the constituent of PM_2.5_ and UF particles with the highest HR and ran additional regressions that included each of the other constituents in the same particle size. The HR and CIs are presented for a change in their respective interquartile ranges (IQRs) unless otherwise noted. Statistical significance was based on a *p*-value < 0.05, and model goodness-of-fit was based on the Akaike information criterion (AIC). The analysis was conducted using PHREG in SAS software (SAS Institute Inc., Cary, NC).

## Results

Of the 133,479 women who completed a baseline questionnaire, we excluded 21,302 with no pollution data (of whom 14,670 had a lack of information on residential addresses and 6,632 lived in areas for which exposure estimates were not available); 1,363 women who had died or moved before the start of follow-up; 406 who were < 30 years of age in January 2001; 4,684 who had unknown or outlier BMI; 3,609 who were missing smoking data; 14 who were excluded because they consented to be included only in breast cancer studies; and 217 who had < 6 months of pollution values during January 2000 through December 2000. The final total was 101,884 women eligible for the study. The average length of follow-up was 6.3 years, with total person-years of 642,269. A total of 6,285 deaths occurred during the follow-up from January 2001 through July 2007; of these, 2,400 were due to cardiovascular diseases, 1,085 were due to IHD, and 929 were due to pulmonary diseases. As indicated in Table 1, the average age of eligible cohort members at the start of follow-up was 57 years, 86% of these women were non-Hispanic white, and 5% were current smokers. Table 2 summarizes the mean and distributions of the concentrations of PM_2.5_ and UF constituents used in the analysis. For example, the mean of PM_2.5_ was 17.9 μg/m^3^ with OC (3.9 μg/m^3^) and nitrate (3.7 μg/m^3^) the largest constituents. For UFs, the mean was 1.3 μg/m^3^ with OC the largest contributor at 0.9 μg/m^3^.

**Table 1:** Descriptive statistics for health and covariate variables for women in the analysis.

Covariate	Percent or mean ± SD
Age at January 2001 (years)	57.3 ± 13.8
Race (% non-Hispanic white)	86.4
Smoking status
Never smoker	68.4
Former smoker	26.9
Current smoker	4.7
Total pack-years	14.7 ± 17.1
Adult secondhand smoke exposure	48
BMI (kg/m^2^)	24.9 ± 5.1
Married/living with partner	46.6
Nondrinker	32.2
Menopausal status and HT use
Premenopausal	41.0
Peri/postmenopausal and no hormone therapy use	11.9
Peri/postmenopausal and current/past hormone therapy use	33.9
Unknown menopausal status/hormone therapy use	13.2
Dietary fat (g/day)	56.3 ± 26.8
Dietary fiber (g/day)	15.2 ± 6.4
Dietary calories (kcal/day)	1595.4 ± 556.4
Physical activity (hr/week)	4.41 ± 4.0
Family history of heart disease	54.4
Taking hypertension medication/aspirin	34.3
All characteristics were reported on baseline questionnaire, except marital status, which was reported on the 2000 questionnaire.	

**Table 2:** Distribution of fine and UF particles species and sources.

	PM_2.5 _(μg/m^3^)				UF (ng/m^3^)			
	Mean	25th	Median	75th	Mean	25th	Median	75th
Pollutant
Mass	17.9	13.1	18.2	22.8	1,293	778	1,214	1,747
Cu	0.5^a^	0.2^*a*^	0.4^*a*^	0.6^*a*^	0.03	0.01	0.01	0.03
Fe	0.4	0.3	0.4	0.5	1.3	0.9	1.3	1.6
Mn	7.7^*a*^	5.7^*a*^	7.9^*a*^	9.8^*a*^	0.05	0.02	0.03	0.05
Nitrate	3.7	1.5	3.5	5.4	—	—	—	—
EC	1.1	0.6	1.0	1.5	113	63	103	156
OC	3.9	2.4	3.7	5.2	908	507	845	1,238
Other compounds	2.9	2.1	2.9	3.6	36	18	29	47
Other metals^*b*^	1.0	0.7	1.0	1.2	21	12	19	28
SOA_bio	0.1	0.1	0.1	0.1	17	9	16	24
SOA_ant	0.1	0.05	0.1	0.1	23	11	23	34
Sources of primary particles
On-road gasoline	0.3	0.2	0.3	0.5	109	49	90	157
Off-road gasoline	0.2	0.1	0.1	0.2	34	16	29	49
On-road diesel	0.4	0.2	0.4	0.6	62	33	58	88
Off-road diesel	1.0	1.0	1.0	1.4	93	53	83	126
Wood smoke	1.4	0.5	0.9	1.8	310	105	205	437
Meat cooking	1.1	0.4	0.8	1.6	115	46	86	174
High-sulfur fuel combustion	0.4	0.1	0.3	0.5	49	10	21	64
Other anthropogenic	7.0	5.2	7.2	9.0	502	253	403	653
25th and 75th are percentiles. ^***a***^Concentrations × 1,000. ^***b***^Metals besides Cu, Fe, and Mn.								

A majority of the species were moderately to highly correlated (*r* = 0.5–0.8) (see Supplemental Material, Tables S1 and S2). PM_2.5_ nitrates had correlations of 0.55, 0.43, 0.65, and 0.84 with EC, OC, Cu, and SOA_ant, respectively. For UFs, EC had correlations of 0.67, 0.19, and 0.40 with OC, Cu, and SO_ant, respectively. The average inter-constituent correlation for PM_2.5_ was 0.59 and for UFs was 0.64. The implications of these correlations are described in “Discussion.”

**Figure 2:**
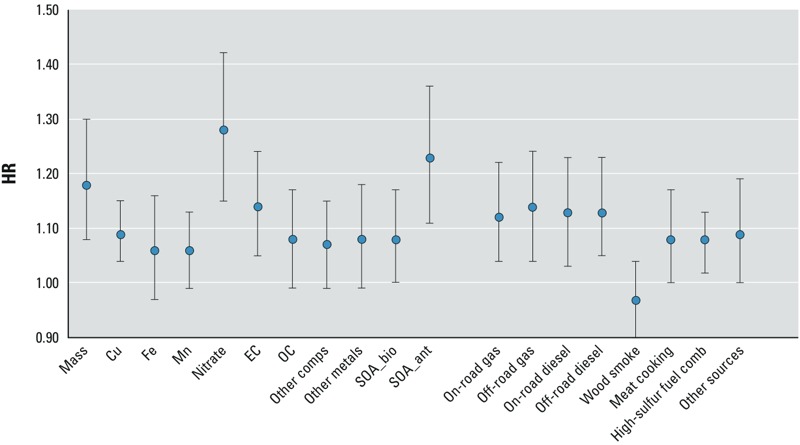
Association of PM_2.5_ constituents and sources with IHD mortality (HRs and 95% CIs using interquartile range. Abbreviations: comb, combustion; comps, components.

In the Cox proportional hazards regression analysis for PM_2.5_, the only statistically significant association (*p* < 0.05) observed between sources and constituents and all-cause mortality was for the source of high-sulfur fuel combustion (HR = 1.03; 95% CI: 1.01, 1.05 for a change in its IQR), and there were no statistically significant associations with pulmonary disease mortality (see Supplemental Material, Table S3). For cardiovascular disease mortality, statistically significant associations were demonstrated only for nitrate (HR = 1.10; 95% CI: 1.02, 1.18) and high-sulfur fuel combustion (HR = 1.05; 95% CI: 1.02, 1.09). Associations with *p*-values < 0.10 were observed for PM_2.5_ mass (HR = 1.05; 95% CI: 0.99, 1.12) and SOA_ant (HR = 1.06; 95% CI: 0.99, 1.13) (see Supplemental Material, Table S3). As summarized in Table 3 and Figure 2, however, there were many statistically significant associations with IHD mortality. Among the constituents, nitrate (HR = 1.28; 95% CI: 1.16, 1.42) and SOA_ant (HR = 1.23; 95% CI: 1.11, 1.36) were the most statistically significant, and both had higher HRs and fit the data slightly better, based on the (lower) AIC, than that of PM_2.5_ mass (HR = 1.18; 95% CI: 1.08, 1.30). Among the emission sources, we found statistically significant associations between IHD and all four of the vehicle sources, meat cooking, and high-sulfur content fuel combustion.

**Table 3:** Hazard ratios (HRs) and 95% CIs for associations of PM_2.5_ and UF particles with IHD Mortality.

	PM_2.5 _(μg/m^3^)				UF (ng/m^3^)			
	IQR	HR^*a*^ (95% CI)	*p*-Value	AIC	IQR	HR^*a*^ (95% CI)	*p*-Value	AIC
Pollutant
Mass	9.6	1.18 (1.08, 1.30)	< 0.001	14,011	969	1.10 (1.02, 1.18)	0.01	13,896
Cu	0.4^*b*^	1.09 (1.04, 1.15)	< 0.001	14,015	0.02	1.06 (1.03, 1.09)	< 0.0001	13,890
Fe	0.2	1.06 (0.97, 1.16)	0.17	14,023	0.8	1.03 (1.00, 1.06)	< 0.05	13,899
Mn	4.0^*b*^	1.06 (0.99, 1.13)	0.12	14,023	0.03	1.00 (0.99, 1.01)	0.62	13,902
Nitrate	3.9	1.28 (1.16, 1.42)	< 0.0001	14,003	—	—		—
EC	0.8	1.14 (1.05, 1.24)	< 0.01	14,015	93	1.15 (1.06, 1.26)	< 0.001	13,891
OC	2.8	1.08 (0.99, 1.17)	0.07	14,022	731	1.08 (1.01, 1.15)	< 0.05	13,898
Other compounds	1.4	1.07 (0.99, 1.15)	0.08	14,022	29	1.10 (1.04, 1.16)	< 0.001	13,892
Other metals^*c*^	0.5	1.08 (0.99, 1.18)	0.09	14,022	17	1.13 (1.05, 1.21)	< 0.01	13,892
SOA_bio	0.1	1.08 (1.00, 1.17)	< 0.05	14,021	14	1.10 (1.02, 1.19)	< 0.01	13,896
SOA_ant	0.1	1.23 (1.11, 1.36)	< 0.0001	14,009	24	1.25 (1.13, 1.39)	< 0.001	13,884
Sources of primary particles
On-road gasoline	0.3	1.12 (1.04, 1.22)	< 0.01	14,017	108	1.12 (1.04, 1.22)	< 0.01	13,894
Off-road gasoline	0.2	1.14 (1.04, 1.24)	< 0.01	14,016	33	1.14 (1.04, 1.24)	< 0.01	13,894
On-road diesel	0.4	1.13 (1.03, 1.23)	< 0.01	14,018	56	1.13 (1.03, 1.24)	< 0.01	13,895
Off-road diesel	0.8	1.13 (1.05, 1.23)	< 0.05	14,015	73	1.14 (1.05, 1.23)	< 0.01	13,892
Wood smoke	1.3	0.97 (0.90, 1.04)	0.38	14,024	332	0.95 (0.89, 1.02)	0.20	13,900
Meat cooking	1.2	1.08 (1.00, 1.17)	< 0.05	14,021	128	1.11 (1.03, 1.20)	< 0.01	13,895
High-sulfur fuel combustion	0.4	1.08 (1.02, 1.13)	< 0.05	14,017	54	1.08 (1.04, 1.12)	< 0.0001	13,888
Other anthropogenic	3.8	1.09 (1.00, 1.19)	0.05	14,021	400	1.06 (1.01, 1.10)	0.01	13,896
^***a***^HRs were stratified for age and race and adjusted for smoking status, smoking pack-years, adult secondhand smoke exposure, BMI, marital status, alcohol consumption, physical activity, menopausal status and HT use combined, family history of heart disease, hypertension medication/aspirin use, and dietary fat, fiber, and caloric intake. ^***b***^Concentrations × 1,000. ^***c***^Metals other than Cu, Fe, and Mn.								

For UFs, no statistically significant associations were observed for either all-cause or pulmonary mortality (see Supplemental Material, Table S4). For cardiovascular mortality, significant associations were noted for Cu (HR = 1.03; 95% CI: 1.00, 1.05), and the sources of high-sulfur fuel combustion (HR = 1.04; 95% CI: 1.01, 1.07). However, many statistically significant associations were again demonstrated for IHD mortality (Table 3). Among the species, this includes Cu, Fe, EC, OC, other compounds and metals, SOA_ant, and SOA_bio. The largest estimated risks were for SOA_ant (HR = 1.25; 95% CI: 1.13, 1.39), EC (HR = 1.15; 95% CI: 1.06, 1.26), and other metals (HR = 1.13; 95% CI: 1.05, 1.21), each of which had lower *p*-values and slightly better fitting models based on AIC than did UF mass (HR = 1.10; 95% CI: 1.02, 1.18). Many of the other constituents also had better model fits than PM_2.5_. Among the sources, associations were seen for both on and off-road diesel and gasoline, meat cooking, high-sulfur fuel combustion, and other anthropogenic sources.

The analysis of IHD mortality showed that although PM_2.5_ mass had a lower *p*-value than UF mass, UF mass and each of the UF constituents provided a better fit and had a lower *p*-value than their corresponding PM_2.5_ constituent (except for Mn, for which there was no statistical significance for either particle size).

In our sensitivity analysis, we found that adding the six contextual variables to the model did not quantitatively alter any of the results (HR or *p*-value) except in one case where PM_2.5_ SOA_bio became nonsignificant (data not shown). We also examined two-pollutant models with the PM_2.5_ constituent with the largest effect estimate for IHD (PM_2.5_ nitrate) in a regression with each of the other PM_2.5_ constituents. Likewise, we examined two-pollutant models for UF (SOA_ant) with each of the other UF constituents (Tables 4 and 5). For the two-pollutant models with PM_2.5_ nitrate, the HRs for nitrate were basically unchanged and none of the other PM_2.5_ constituents, including mass, were statistically significant. For UFs SOA_ant, the HR was again basically unchanged and only one other constituent, Cu, was also statistically significantly related to IHD mortality.

**Table 4:** Hazard ratios (HR) and 95% CIs for ischemic heart disease mortality for two-pollutant models of PM_2.5_ nitrate with each of the other constituents.

Pollutant	PM_2.5_ constituent (μg/m^3^)			PM_2.5_ nitrate (μg/m^3^)		
	IQR	HR^*a*^ (95% CI)	*p*-Value	IQR	HR^*a*^ (95% CI)	*p*-Value
Mass	9.6	1.03 (0.91, 1.18)	0.61	3.9	1.25 (1.07, 1.45)	< 0.05
Cu	0.4^*b*^	1.02 (0.94, 1.10)	0.67	3.9	1.26 (1.11, 1.44)	< 0.001
Fe	0.2	0.92 (0.82, 1.03)	0.14	3.9	1.35 (1.19, 1.54)	< 0.0001
Mn	4.0^*b*^	0.94 (0.85, 1.04)	0.23	3.9	1.34 (1.18, 1.53)	< 0.0001
Nitrate	—	—	—	3.9	1.28 (1.16, 1.42)	< 0.0001
EC	0.8	1.04 (0.94, 1.14)	0.49	3.9	1.25 (1.11, 1.42)	< 0.001
OC	2.8	1.00 (0.91, 1.09)	0.94	3.9	1.29 (1.15, 1.44)	< 0.0001
Other compounds	1.4	0.96 (0.87, 1.05)	0.34	3.9	1.33 (1.17, 1.51)	< 0.0001
Other metals^*c*^	0.5	0.93 (0.83, 1.04)	0.21	3.9	1.35 (1.18, 1.53)	< 0.0001
SOA_bio	0.1	0.95 (0.86, 1.05)	0.31	3.9	1.34 (1.17, 1.53)	< 0.0001
SOA_ant	0.1	0.97 (0.78, 1.21)	0.78	3.9	1.32 (1.05, 1.66)	0.02
^***a***^HRs were stratified for age and race and adjusted for smoking status, smoking pack-years, adult secondhand smoke exposure, BMI, marital status, alcohol consumption, physical activity, menopausal status and HT use combined, family history of heart disease, hypertension medication/aspirin use, and dietary fat, fiber, and caloric intake. ^***b***^Concentrations × 1,000. ^***c***^Metals other than Cu, Fe, and Mn.						

**Table 5:** Hazard ratios (HR) and 95% CIs for ischemic heart disease mortality for two-pollutant models of anthropogenic UF secondary organic aerosols with each of the other constituents.

Pollutant	UF constituent (ng/m^3^)			UF SOA_ant (ng/m^3^)		
	IQR	HR^*a*^ (95% CI)	*p*-Value	IQR	HR^*a*^ (95% CI)	*p*-Value
Mass	969	1.03 (0.94, 1.12)	0.56	24	1.19 (1.08, 1.31)	< 0.001
Cu	0.02	1.39 (1.05, 1.83)	0.02	24	1.16 (1.06, 1.28)	0.001
Fe	0.8	1.01 (0.97, 1.06)	0.63	24	1.20 (1.09, 1.31)	< 0. 001
Mn	0.03	1.00 (0.99, 1.01)	0.95	24	1.21 (1.11, 1.32)	< 0.0001
EC	93	1.04 (0.93, 1.16)	0.52	24	1.18 (1.05, 1.32)	0.006
OC	731	1.02 (0.95, 1.10)	0.61	24	1.20 (1.09, 1.31)	< 0.001
Other compounds	29	1.06 (1.00, 1.13)	0.06	24	1.17 (1.07, 1.29)	< 0.001
Other metals^*b*^	17	1.07 (0.96, 1.18)	0.22	24	1.17 (1.06, 1.29)	0.002
SOA_bio	14	0.99 (0.92, 1.07)	0.82	24	1.22 (1.09, 1.36)	< 0.001
SOA_ant	—	—	—	24	1.25 (1.13, 1.39)	< 0.001
^***a***^HRs were stratified for age and race and adjusted for smoking status, smoking pack-years, adult secondhand smoke exposure, BMI, marital status, alcohol consumption, physical activity, menopausal status and HT use combined, family history of heart disease, hypertension medication/aspirin use, and dietary fat, fiber, and caloric intake. ^***b***^Metals other than Cu, Fe, and Mn.						

## Discussion

Our analysis of long-term exposure to the mass and constituents of PM_2.5_ and UF particles revealed several statistically significant associations with all-cause, cardiovascular, and IHD mortality. For PM_2.5_, high-sulfur content fuel combustion was associated with all three end points, and nitrates were associated with cardiovascular and IHD mortality. Several other constituents reached statistical significance with IHD mortality including PM_2.5_ mass, Cu, EC, and the SOAs, as well as the sources including gas- and diesel-fueled vehicles, meat cooking, and high-sulfur fuel combustion. Among the PM_2.5_ constituents, based on their associated IQRs, nitrate had the highest HR and provided the best fit of the data. For UFs, constituents such as SOA_ant, EC, and “other” metals exhibited statistically significant associations with IHD mortality, as did all of the mobile sources and high-sulfur fuel combustion. For both PM_2.5_ and UF particles, several constituents generated higher HRs based on their relevant IQRs than their associated mass measurements and in some cases (e.g., UF mass vs. SOA_ant) the differences were statistically significant based on methodology suggested by Schenker and Gentleman (2001). In addition, for all of the constituents, there were better model fits, based on AIC, for UFs than for PM_2.5_.

In a previous analysis of the CTS (based on 73,489 women), exposures to PM_2.5_ were estimated using data from 77 existing monitors located throughout the state (Lipsett et al. 2011). Smoothed surfaces were produced through inverse distance weighting, and grids of 250 m were created. Monthly concentrations were assigned to residents within each grid with the added constraint that participants were required to be within 30 km of a monitor. That study produced an HR for the association of PM_2.5_ and IHD mortality of 1.20 (95% CI: 1.02, 1.41) for a 10-μg/m^3^ increase in PM_2.5_. This result comports with the HR estimate (converted to 10 μg/m^3^ change) in the present study of 1.19 (95% CI: 1.08, 1.31). Our estimate is also similar to those for IHD mortality based on analyses of the American Cancer Society (ACS) cohort in which the HRs for a 10-μg/m^3^ increase in PM_2.5_ were 1.18 (95% CI: 1.14, 1.23) for the United States and 1.11 (95% CI: 1.05, 1.18) for California (Jerrett et al. 2013; Pope et al. 2002). They were also comparable to those of the Harvard Six Cities Study of 1.26 (95% CI: 1.08, 1.47) (Laden et al. 2006) for a 10-μg/m^3^ increase in PM_2.5_.

We can also compare the estimates of a few constituents of PM_2.5_ with those obtained in a prior analysis of a smaller subset (*n* = 43,220) of the CTS (Ostro et al. 2010). In this prior analysis, we used a 30-km buffer catchment area around each of eight U.S. EPA Speciation Trends Network monitors. The HR for cardiovascular mortality associated with a 1-μg/m^3^ increase in nitrate in the previous study was 1.03 (95% CI: 1.01, 1.06) versus the present study estimate of 1.02 (95% CI: 1.01, 1.04). For EC, the previous study generated an HR of 1.11 (95% CI: 0.91, 1.36) for a 1-μg/m^3^ change compared with 1.05 (95% CI: 0.98, 1.11) in the present study. Several cohort studies have estimated the effects of EC or its correlates on cardiovascular mortality. For example, Smith et al. (2009) estimated its effects among 352,000 participants in the ACS cohort and reported a relative risk (RR) of 11% (95% CI: 3, 19) per 1 μg/m^3^. The estimated RR of coronary heart disease mortality associated with EC was 1.08 (95% CI: 1.04, 1.12) per 1 μg/m^3^ in a cohort study in Vancouver, Canada (Gan et al. 2011). In addition, the RR of cardiovascular mortality from long-term exposure to black smoke, another EC correlate which measures the light reflectance of particles was reported in cohort studies in the Netherlands and Scotland (Beelen et al. 2008; Beverland et al. 2012). Based on a conversion factor calculated by Janssen et al. (2011), the HRs were 1.04 (95% CI: 0.95, 1.12) and 1.06 (95% CI: 1.0, 1.11) per μg/m^3^ of EC, respectively. Finally, a recent study (Lippman et al. 2013) examined the effect of PM_2.5_ components and sources using a subset of the national ACS cohort. The results of the Cox regression model for IHD were generally supportive of our findings. Among the components measured, the authors observed statistically significant associations with IHD for EC and several of the metals (e.g., iron, lead, nickel, and zinc). Nitrates were not included in the ACS study, but statistical associations were observed for sulfur, likely from the combustion of coal and residual oil, which was not included in our study. In addition, among the sources, traffic was dominant in both studies.

We did not observe any positive associations with long-term exposure to wood smoke, although associations of short-term exposure with respiratory outcomes have been reported (Naeher et al. 2007). This may be attributable to the episodic nature of the wood smoke or to possible confounding by socioeconomic status. In California, most of the population-weighted exposure occurred in relatively high-income counties, such as San Francisco, San Mateo, and Santa Clara, where greater longevity prevails.

Given its large spatial variability, assessing exposure to UF particles among participants in cohort studies has been challenging. Therefore, very few studies have measured or estimated long-term exposures to UFs at a fine enough spatial gradient to examine its impact on health. As an alternative, several studies have attempted to estimate the effects of exposure to traffic, often a major source of UFs, using metrics such as nitrogen dioxide, distance to major roadways, and/or local traffic density (Health Effects Institute 2010). In general, within the first 250 m or so of a major roadway, UFs may be highly correlated with other pollutants such as black carbon, nitrogen dioxide, and carbon monoxide. However, the relation between UFs and these other pollutants, especially away from major roadways, is not precise and the correlations may be fairly low (Sioutas et al. 2005; Zhu et al. 2008).

In contrast, several studies have estimated the effects of daily changes in UFs where only the time-varying component is needed (Atkinson et al. 2010; Forastiere et al. 2005; Peters et al. 2009). The previous studies were based on counts of UFs rather than mass, so their estimates are not directly comparable to ours. In support of these and our findings on UFs, Delfino et al. (2009) followed a panel of 60 elderly subjects with coronary artery disease and reported associations between biomarkers of inflammation and several components of UF particles, including EC and primary OC. Other animal and human studies have implicated transition metals in generating inflammation and oxidative stress (Chen and Lippmann 2009; Costa and Dreher 1997; Gurgueira et al. 2002).

Our study has both strengths and limitations. Among the strengths are the relatively large size of the cohort, the low prevalence of active smoking, and the relative similarity of occupational status and activity patterns. These factors all help to reduce residual confounding in our estimates. Second, the study population included a large number of women at risk of developing cardiovascular disease by virtue of their age and postmenopausal status. Third, because of the level of spatial detail in the pollution estimates and the information on residential history, the temporal and spatial resolution of the pollution exposure is enhanced relative to many previous cohort studies.

One limitation is that the study was restricted to women, and these women were not necessarily representative of all women. Second, only about 1,000 women were diagnosed with IHD or pulmonary mortality, which may introduce some instability in the risk estimates. Third, our estimates could be impacted by possibly correlated and unmeasured co-pollutants. Fourth, there was high intercorrelation (most between 0.5 and 0.8) among the particle constituents, different levels of uncertainty and bias in their modeled estimates, and potentially different exposure patterns. These factors could affect the estimates of their relative toxicity. The high correlations reflect *a*) a consistent chemical signature of multiple pollutants associated with PM emitted from major sources, *b*) that some elements are dominated by a small number of sources, and/or *c*) the similarity of certain pollutants from different sources such as gasoline and diesel vehicles. A similar range of intercorrelation among the constituents was reported by Ostro et al. (2010), which used monitored values for the same cohort as the present study, but only included eight metropolitan areas. Thus the high correlations are not simply a result of the modeling methodology. However, this feature does make it difficult to identify unique components and sources that are associated with adverse health effects. Fifth, stationary sources contribute < 15% of the PM_2.5_ in California (Air Resources Board 2013), so sources such as coal burning and industrial processes and their specific constituents are not included in this study. Finally, although our exposure method had some significant enhancements over previous assessments, some misclassification will continue to exist.

Nevertheless, this study represents an innovative effort to estimate the effects of long-term exposure to the constituents of two pollutants, fine and ultrafine particles, that are ubiquitous in our environment. As such, it provides evidence of the public health impact of a subset of these constituents and helps contribute to our understanding of air pollution–related cardiovascular disease.

## Supplemental Material

(491 KB) PDF
