# Supplementary material for: Associations of Mortality with Long-Term Exposures to Fine and Ultrafine Particles, Species and Sources: Results from the California Teachers Study Cohort
Source: Environ Health Perspect. 2015 Jan 23;123(6):549–56. doi: 10.1289/ehp.1408565 (PMC4455590; doi:10.1289/ehp.1408565)
Supplement: (491 KB) PDF [file ehp.1408565.s001.508.pdf]

Environmental Health Perspectives doi:10.1289/ehp.1408565

Supplemental Material

Associations of Mortality with Long-Term Exposures to Fine and Ultrafine Particles, Species and Sources: Results from the California Teachers Study Cohort

Bart Ostro, Jianlin Hu, Debbie Goldberg, Peggy Reynolds, Andrew Hertz, Leslie Bernstein, and Michael J. Kleeman

Table S1. Correlations between $PM_{2.5}$ and various chemical species calculated from model predictions.

Exposure	Mass	Cu	EC	Fe	Mn	Oth Mtl	Nit	OC	Oth Spc	S_ant	S_bio
Mass	-										
Cu	0.64	-									
EC	0.74	0.44	-								
Fe	0.86	0.69	0.58	-							
Mn	0.83	0.74	0.55	0.96	-						
Oth Mtl	0.85	0.68	0.56	1.00	0.95	-					
Nit	0.81	0.65	0.55	0.80	0.79	0.81	-				
OC	0.73	0.38	0.90	0.52	0.49	0.49	0.43	-			
Oth Spc	0.83	0.70	0.48	0.97	0.94	0.98	0.80	0.42	-		
S_ant	0.76	0.51	0.54	0.62	0.61	0.61	0.84	0.42	0.59	-	
S_bio	0.62	0.53	0.38	0.65	0.65	0.65	0.75	0.33	0.66	0.79	-

Oth Mtl = Other metals; Oth Spc = Other species; Nit = Nitrate; S_ant = anthropogenic; S_bio = biogenic secondary organics.

Table S2. Correlations between UF and various chemical species calculated from model predictions.

Exposure	Mass	Cu	EC	Fe	Mn	Oth Mtl	Nit	OC	Oth Spc	S_ant	S_bio
Mass	-										
Cu	0.48	-									
EC	0.74	0.19	-								
Fe	0.77	0.64	0.69	-							
Mn	0.27	0.50	0.19	0.63	-						
Oth Mtl	0.94	0.71	0.70	0.86	0.34	-					
Nit	-	-	-	-	-	-	-				
OC	0.99	0.48	0.67	0.73	0.25	0.42	-	-			
Oth Spc	0.71	0.87	0.62	0.81	0.40	0.93	-	0.68	-		
S_ant	0.64	0.37	0.40	0.53	0.20	0.88	-	0.62	0.41	-	
S_bio	0.70	0.22	0.42	0.56	0.15	0.62	-	0.69	0.34	0.75	-

Oth Mtl = Other metals; Oth Spc = Other species; Nit = Nitrate; S_ant = anthropogenic; S_bio = biogenic secondary organics.

Table S3. Hazard ratios (HR) and 95% confidence interval (CI) for association of $PM_{2.5}$ and mortality outcomes.

Exposure	IQR ($\mu g/m^3$)	All-cause mortality HR[a] (95% CI)	p-value	Cardiovascular mortality HR[a] (95% CI)	p-value	Pulmonary mortality HR[a] (95% CI)	p-value
**Pollutant**							
Mass	9.6	1.01 (0.98, 1.05)	0.47	1.05 (0.99, 1.12)	0.10	0.99 (0.90, 1.09)	0.87
Cu	0.4[b]	1.00 (0.98, 1.03)	0.77	1.02 (0.98, 1.06)	0.32	1.00 (0.94, 1.08)	0.92
Fe	0.2	1.00 (0.97, 1.04)	0.83	1.00 (0.95, 1.06)	0.93	0.99 (0.90, 1.09)	0.87
Mn	4.0[b]	1.00 (0.96, 1.03)	0.80	1.00 (0.95, 1.06)	0.98	0.98 (0.90, 1.08)	0.72
Nitrate	3.9	1.03 (0.99, 1.08)	0.13	1.10 (1.02, 1.18)	<0.01	1.02 (0.90, 1.14)	0.80
EC	0.8	1.00 (0.97, 1.04)	0.95	1.04 (0.98, 1.09)	0.22	1.00 (0.91, 1.09)	0.96
OC	2.8	1.00 (0.97, 1.04)	0.92	1.01 (0.96, 1.07)	0.66	0.99 (0.90, 1.09)	0.83
Other compounds	1.4	1.01 (0.98, 1.04)	0.46	1.01 (0.96, 1.06)	0.61	1.01 (0.93, 1.09)	0.85
Other metals[c]	0.5	1.01 (0.97, 1.05)	0.63	1.01 (0.95, 1.07)	0.69	1.00 (0.90, 1.10)	0.92
SOA biogenic	0.1	1.03 (0.99, 1.06)	0.10	1.04 (0.98, 1.09)	0.18	1.05 (0.96, 1.15)	0.26
SOA anthropogenic	0.1	1.02 (0.98, 1.06)	0.41	1.06 (0.99, 1.13)	0.10	1.00 (0.89, 1.12)	0.99
**Sources of primary particles**							
On-road gasoline	0.3	0.99 (0.95, 1.02)	0.46	1.01 (0.96, 1.07)	0.60	0.94 (0.86, 1.03)	0.21
Off-road gasoline	0.2	0.99 (0.96, 1.03)	0.65	1.03 (0.97, 1.09)	0.31	0.97 (0.88, 1.07)	0.58
On-road diesel	0.4	1.00 (0.97, 1.04)	0.87	1.02 (0.96, 1.09)	0.47	0.98 (0.88, 1.08)	0.67
Off-road diesel	0.8	1.00 (0.97, 1.04)	0.98	1.04 (0.98, 1.09)	0.20	1.00 (0.92, 1.10)	0.97
Wood smoke	1.3	1.01 (0.98, 1.04)	0.36	0.99 (0.95, 1.04)	0.71	1.02 (0.94, 1.10)	0.63
Meat cooking	1.2	0.98 (0.95, 1.02)	0.33	1.01 (0.96, 1.07)	0.73	1.00 (0.92, 1.09)	0.97
High sulfur fuel combustion	0.4	1.03 (1.01, 1.05)	<0.005	1.05 (1.02, 1.09)	<0.004	1.01 (0.95, 1.07)	0.75
Other anthropogenic	3.8	1.01 (0.97, 1.04)	0.76	1.01 (0.95, 1.07)	0.75	0.99 (0.90, 1.09)	0.85

[a]HRs stratified for age and race and adjusted for smoking status, smoking pack-years, adult second-hand smoke exposure, BMI, marital status, alcohol consumption, physical activity, menopausal status and HT use combined, family history of heart disease, hypertension medication/aspirin use, dietary fat, fiber and caloric intake. [b]Concentrations x1000. [c]Metals other than Cu, Fe, and Mn.

Table S4. Hazard ratios (HR) and 95% confidence interval (CI) for association of UF and mortality outcomes.

Exposure	IQR ($\mu g/m^3$)	All-cause mortality HR[a] (95% CI)	p-value	Cardiovascular mortality HR[a] (95% CI)	p-value	Pulmonary mortality HR[a] (95% CI)	p-value
**Pollutant**							
Mass	969	1.01 (0.98, 1.05)	0.38	1.03 (0.97, 1.08)	0.33	1.01 (0.93, 1.10)	0.82
Cu	0.02	1.00 (0.99, 1.02)	0.89	1.03 (1.00, 1.05)	0.02	0.98 (0.93, 1.02)	0.29
Fe	0.8	1.00 (0.98, 1.02)	0.98	1.01 (0.98, 1.04)	0.53	0.99 (0.93, 1.06)	0.76
Mn	0.03	1.00 (0.99, 1.00)	0.31	1.00 (0.99, 1.01)	0.80	0.99 (0.96, 1.02)	0.45
Nitrate	-	-		-	-	-	-
EC	93	1.01 (0.97, 1.04)	0.72	1.04 (0.98, 1.10)	0.20	1.01 (0.92, 1.11)	0.88
OC	731	1.01 (0.98, 1.04)	0.43	1.02 (0.97, 1.07)	0.47	1.01 (0.93, 1.09)	0.83
Other compounds	29	1.00 (0.98, 1.03)	0.71	1.04 (1.00, 1.08)	0.05	0.98 (0.91, 1.05)	0.52
Other metals[b]	17	1.01 (0.98, 1.04)	0.51	1.04 (0.99, 1.09)	0.14	0.98 (0.90, 1.07)	0.63
SOA biogenic	14	1.03 (1.00, 1.07)	0.08	1.04 (0.99, 1.10)	0.12	1.06 (0.97, 1.15)	0.21
SOA anthropogenic	24	1.02 (0.98, 1.07)	0.29	1.07 (0.99, 1.14)	0.07	1.01 (0.90, 1.13)	0.84
**Sources of primary particles**							
On-road gasoline	108	0.99 (0.95, 1.02)	0.49	1.02 (0.96, 1.07)	0.59	0.94 (0.86, 1.03)	0.21
Off-road gasoline	33	0.99 (0.96, 1.03)	0.69	1.03 (0.97, 1.09)	0.31	0.98 (0.88, 1.08)	0.63
On-road diesel	56	1.01 (0.97, 1.05)	0.75	1.02 (0.96, 1.09)	0.44	0.98 (0.89, 1.08)	0.70
Off-road diesel	73	1.00 (0.97, 1.04)	0.91	1.04 (0.98, 1.09)	0.21	1.01 (0.92, 1.10)	0.89
Wood smoke	332	1.01 (0.99, 1.05)	0.33	0.98 (0.94, 1.03)	0.53	1.02 (0.95, 1.10)	0.59
Meat cooking	128	0.99 (0.95, 1.02)	0.42	1.02 (0.97, 1.08)	0.47	0.99 (0.91, 1.08)	0.87
High sulfur fuel combustion	54	1.01 (0.99, 1.03)	0.25	1.04 (1.01, 1.07)	<0.005	0.99 (0.94, 1.04)	0.73
Other anthropogenic	400	1.01 (0.99, 1.03)	0.39	1.02 (0.99, 1.05)	0.30	1.01 (0.96, 1.06)	0.75

[a]HRs stratified for age and race and adjusted for smoking status, smoking pack-years, adult second-hand smoke exposure, BMI, marital status, alcohol consumption, physical activity, menopausal status and HT use combined, family history of heart disease, hypertension medication/aspirin use, dietary fat, fiber and caloric intake. [b]Metals other than Cu, Fe, and Mn.
